# Changes in the Proliferation of the Neural Progenitor Cells of Adult Mice Chronically Infected with *Toxoplasma gondii*

**DOI:** 10.3390/microorganisms11112671

**Published:** 2023-10-31

**Authors:** Verónica Anaya-Martínez, Jhony Anacleto-Santos, Ricardo Mondragón-Flores, Armando Zepeda-Rodríguez, Brenda Casarrubias-Tabarez, Teresa de Jesús López-Pérez, Mariana Citlalli de Alba-Alvarado, Cintli Martínez-Ortiz-de-Montellano, Elba Carrasco-Ramírez, Norma Rivera-Fernández

**Affiliations:** 1 Centro de Investigación en Ciencias de la Salud, Facultad de Ciencias de la Salud, Universidad Anáhuac, Lomas Anáhuac, Naucalpan de Juárez 52786, Estado de México, Mexico; veronica.anaya@anahuac.mx; 2 Departamento de Microbiología y Parasitología, Facultad de Medicina, Universidad Nacional Autónoma de México (UNAM), Coyoacán, Ciudad de México 04510, Mexico; anacletosantosjhony@facmed.unam.mx (J.A.-S.); tere.lopez82@comunidad.unam.mx (T.d.J.L.-P.); marianadealba@comunidad.unam.mx (M.C.d.A.-A.); ramirezce@comunidad.unam.mx (E.C.-R.); 3 Departamento de Bioquímica, CINVESTAV Zacatenco, Ciudad de México 07360, Mexico; rmflores@cinvestav.mx; 4 Departamento de Biología Celular y Tisular, Facultad de Medicina, Universidad Nacional Autónoma de México (UNAM), Coyoacán, Ciudad de México 04510, Mexico; azr@unam.mx (A.Z.-R.); bcasarrubias@facmed.unam.mx (B.C.-T.); 5 Departamento de Parasitología, Facultad de Medicina Veterinaria y Zootecnia, Universidad Nacional Autónoma de México (UNAM), Ciudad de México 04510, Mexico; cintli@unam.mx

**Keywords:** *Toxoplasma gondii*, neural progenitor cells, neurogenesis, chronic toxoplasmosis

## Abstract

During *Toxoplasma gondii* chronic infection, certain internal factors that trigger the proliferation of neural progenitor cells (NPCs), such as brain inflammation, cell death, and changes in cytokine levels, are observed. NPCs give rise to neuronal cell types in the adult brain of some mammals. NPCs are capable of dividing and differentiating into a restricted repertoire of neuronal and glial cell types. In this study, the proliferation of NPCs was evaluated in CD-1 adult male mice chronically infected with the *T. gondii* ME49 strain. Histological brain sections from the infected mice were evaluated in order to observe *T. gondii* tissue cysts. Sagittal and coronal sections from the subventricular zone of the lateral ventricles and from the subgranular zone of the hippocampal dentate gyrus, as well as sagittal sections from the rostral migratory stream, were obtained from infected and non-infected mice previously injected with bromodeoxyuridine (BrdU). A flotation immunofluorescence technique was used to identify BrdU+ NPC. The scanning of BrdU+ cells was conducted using a confocal microscope, and the counting was performed with ImageJ^®^ software (version 1.48q). In all the evaluated zones from the infected mice, a significant proliferation of the NPCs was observed when compared with that of the control group. We concluded that chronic infection with *T. gondii* increased the proliferation of NPCs in the three evaluated zones. Regardless of the role these cells are playing, our results could be useful to better understand the pathogenesis of chronic toxoplasmosis.

## 1. Introduction

During an infection with *Toxoplasma gondii*, the host immune response stimulates the production of different molecules, such as interferon gamma (IFNγ), tumor necrosis factor (TNFα), and nitric oxide. These molecules and other molecular mechanisms not well characterized are involved in the differentiation of tachyzoites into bradyzoites and in the transformation of the host cells into tissue cysts [1,2]. Tissue cysts are located mostly in the brain and remain viable for the lifetime of the host and are responsible for chronic infections [3]. Interconversion from tachyzoites to bradyzoites residing into tissue cysts is regulated by a multilayered cascade of steps involving promoter-based cis-elements, epigenetic modifications, and protein translation control through eukaryotic initiation factor-2 (eIF2) [4,5,6]. Though the interconversion can be induced in vitro by cellular stress with different methods such as alkaline pH (8.1), heat shock, chemical stress (treatment with IFNγ), nutrient deprivation, or mitochondrial inhibitors, in vivo, it is possible that fever (heat shock) and host immune response contribute to the process. Some studies have shown that interconversion could be due to the slow growth of bradyzoites, despite cellular stress [4,7]. Recently, it has been reported that bradyzoites within tissue cysts are physiologically active; therefore, this can explain the correlations between chronic infections and brain damage [8]. It is well known that neuronal damage as well as brain inflammation are factors that influence the proliferation of neural progenitor cells (NPCs) in the brains of some adult mammals [9,10,11]. During chronic toxoplasmosis, brain inflammation and neuronal death can occur [12,13,14].

**Figure 1:**
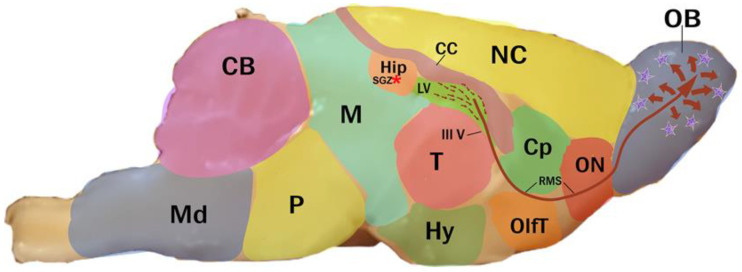
Sagittal image of an adult mouse brain showing the neurogenesis pathway in the subventricular zone and subgranular zone. NPCs are formed in the subventricular zone of the lateral ventricles and migrate to the olfactory bulb through the rostral migratory stream (red asterix). They then migrate to the granular cell layer, where they can develop into functional neurons if they survive. CB—cerebellum, CC—corpus callosum, Cp—caudate putamen, NC—neocortex, Hip—hippocampus, Hy—hypothalamus, M—midbrain, Md—medulla oblongata, OB—olfactory bulb, OlfT—olfactory tubercle, ON—olfactory nucleus, P—pons, T—thalamus, LV—lateral ventricle, III V—third ventricle, RMS—rostral migratory stream, SGZ—hippocampal subgranular zone. Blue stars indicate new neurons.

In rodents, NPC proliferation has been observed in the subgranular zone (SGZ) of the hippocampal dentate gyrus and in the subventricular zone (SVZ) of the lateral ventricles [15,16]. The cells generated in the SVZ migrate through the rostral migratory stream (RMS) to reach the olfactory bulb (OB), which participates in the modulation of olfactory processes. The NPCs generated at the SGZ reach the granular layer of the dentate gyrus, which participates in the regulation of cognitive function (Figure 1) [9,17].

Currently, the restorative potential of NPCs in the damaged brain of adult mammals is unknown. Some authors have observed that there is a migration of NPCs to damaged zones in the mouse brain [18,19,20,21,22,23]. Xiaofeng et al. (2016) showed that in in vitro studies, excretion/secretion antigens of the *T. gondii* RH strain modified the differentiation of C17.2 cells, down-regulating the protein levels of βIII-tubulin [24]. Until now, there are no studies regarding the effect of chronic infection with *T. gondii* on mice NPC proliferation. Therefore, in the present study, this phenomenon was evaluated, since *T. gondii* chronic infection generates several factors in the host contributing to the proliferation of NPCs.

## 2. Materials and Methods

### 2.1. Animals

Animals were obtained from the School of Medicine, UNAM vivarium. For each experiment, ten 8-week-old CD-1 male mice were used. Management was performed according to the Mexican Official Norm NOM-062-ZOO-1999 [25] for the production, care, and use of laboratory animals in accordance with international guidelines. Animal management was approved by the ethics School of Medicine UNAM’s Ethics Committee (project 052/2017). Mice were divided into two groups of ten mice each. Although both outbred and inbred strains of laboratory mice are relatively susceptible to *T. gondii* infection, inbred mice such as BALB/c are resistant to latent infection because of their MHC haplotype (D), whereas CD-1 mice are susceptible [26]; additionally, the CD-1 strain provides a powerful experimental platform for appropriately accounting for the background genetic variation by which human disease manifests [27].

### 2.2. Parasites

The ME49 *T. gondii* strain was kindly provided by the Department of Biochemistry CINVESTAV, Zacatenco Mexico. It was used and maintained by serial passages in CD-1 mice.

### 2.3. Purification of ME49 T. gondii Tissue Cysts and Mouse Infection

Tissue cysts were obtained from the brains of 10 previously infected mice with an oral dose of 10 cysts per animal. Animals were euthanized 12 weeks after infection by administration of a lethal dose of sodium pentobarbital (200 mg/kg intraperitoneally). The brain was removed at necropsy and then macerated in 2 mL of PBS using a glass homogenizer. The macerated tissue samples were observed under light microscopy to corroborate the presence of *T. gondii* cysts. Cyst images were taken with Image Pro 7^®^ (Media Cybernetics, Rockville, MD, USA) software for Windows. The brain and cyst suspensions were filtered using double gauze to remove the larger tissue remains, and the filtrate was then centrifuged at 500 rpm for 5 min. Small aliquots of the suspension were placed in a glass Petri dish, and the cysts were collected using a stereoscopic microscope and a Pasteur pipette. Purified cysts were placed in a 50 µL Eppendorf tube with PBS [28].

### 2.4. BrdU Administration and Preparation of Coronal and Sagittal Sections of the Subventricular Zone, Subgranular Dentate Gyrus Zone, and Rostral Migratory Stream

Ten mice were infected with 10 cysts per animal by oral gavage according to the method described by Haroon et al. (2012), and 10 mice were maintained as the non-infected control group (PBS-mock-infected) [28,29]. Then, 12 weeks after infection and before being euthanized, mice were injected intraperitoneally with two doses of 50 mg/kg of BrdU (spaced 12 h apart), which is the gold standard to evaluate NPC proliferation, since it is incorporated into dividing cells during DNA synthesis and passed down to daughter cells following administration [30,31]. Twelve hours after the last injection of BrdU, mice were anesthetized with sodium pentobarbital and perfused via the aorta with saline and then with 4% paraformaldehyde in 0.1 M phosphate buffer pH 7.4. Brains were then obtained at necropsy and fixed for 24 h in 4% paraformaldehyde, rinsed with PBS, and conserved in 30% saccharose in PBS at 4 °C [29]. The brains from five infected and five non-infected mice were randomly cut in 50 µm sagittal sections from the SVZ, SGZ, and RMS. Coronal sections were obtained from the remaining infected (n = 5) and non-infected (n = 5) animals from the same zones. Sections were obtained with a cryostat and subjected to the flotation immunofluorescence technique for BrdU+. For each zone, 20 sections were obtained.

### 2.5. Histology Process

To identify *T. gondii* tissue cysts in the mice, brain sections from all the infected and non-infected animals were obtained and processed for histopathology and stained with hematoxylin and eosin [32]. Random histological examinations were performed via observation of not less than 10 fields per sample per mouse (in infected and non-infected mice), and images were obtained with Pro Image^®^ software (version 11) for Windows.

### 2.6. Flotation Immunofluorescence Technique for the Identification of NPC BrdU+

Tissue sections were placed in a 12-well plate and permeabilized with 0.5% PBS-Triton X-100 (PBS-T). Later, they were incubated in 1 N HCl for 20 min at 4 °C and then for 30 min at 37 °C, followed by 2 N HCl for 20 min at room temperature. The tissues were rinsed in borate buffer (boric acid) 0.1 M pH 8.5 and then permeabilized with PBS-T 0.5%. Blocking was achieved with normal horse serum (NHS) plus 0.1 M of glycine in PBS-T for 60 min at room temperature and then rinsed with PBS. The tissues were incubated with the primary antibody (α-BrdU monoclonal made in sheep, 1/500 Sigma-Aldrich, St. Louis, MO, USA) in PBS-T 0.5% plus NHS (2%) for 48 h at 4 °C and then washed with PBS-T 0.025%. The tissues were incubated with the secondary antibody (IgG anti-sheep SCBT lab) made in donkey and conjugated to Alexa 488 SCBT Inc. (Santa Cruz Biotechnology, Santa-Cruz, CA, USA) (1/200) in PBS-T 0.5% plus NHS (2%) and then rinsed with PBS 0.1 M. Subsequently, the tissue was mounted on glass slides and covered with Vectashield^®^ (Vector Laboratories, Newark, CA, USA) and coverslips for subsequent scanning [29].

### 2.7. Scanning and Counting of BrdU+ Cells

The areas of interest were scanned using an SP2 Leica^®^ (Leica microsystems, Wetzlar, Germany) confocal microscope, and then 20 optical sections from each zone (1.5 μm optical thickness, total 40–45 μm for each slice in the z-series/per mouse) were analyzed using an excitation wavelength of 488 nm, with an acquired emission of 519 nm. The fluorescent images were overlapped on the screen monitor using green for fluorescein (BrdU+ cells). The same selection criteria were adapted for infected and non-infected animals. The optical sections were projected to form an image that served as the basis to conduct the cell count. Cells were counted per mm^3^ calculated from the different zones’ length and width with the help of ImageJ^®^ software, which automatically counted cells based on the previously established intensity pixels [33]. This software can determine the specific color or rank to be in the image, and the cell diameter can be assigned; hence, when both criteria are met, the object is counted. In this study, the marked cells measured around 10 µm, and the software searched pixeled areas with a darker tone (bright green); therefore, the stains were counted and identified as a cell. As aforementioned, this software locates the area with a specific type of pixel (bright green), but if the pixeled area is over 10 µm, the program marks it so that it may be manually corrected to decide how many cells there are or whether there are two or more cells together.

Both the coronal and sagittal sections from the SGZ and SVZ were analyzed in order to obtain representative samples that allowed for meaningful statistical evaluations [34]. RMS counting was performed only on sagittal sections, because only in these sections is it possible to observe the whole migratory zone, even though in coronal sections of the anterior subventricular zone of the lateral ventricles, the start of cell migration is commonly observed.

**Figure 2:**
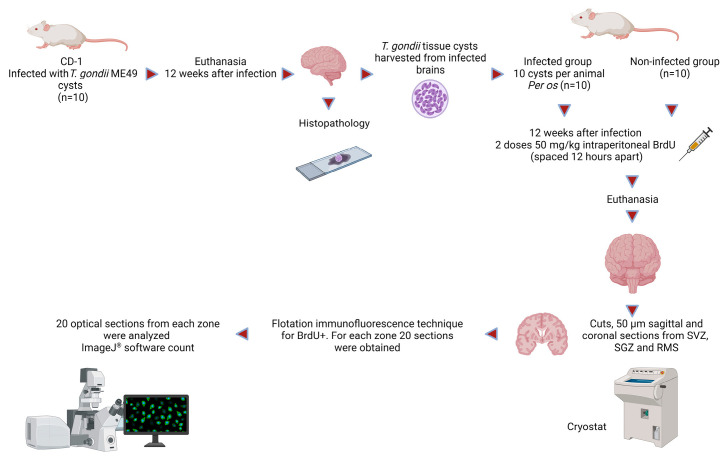
Diagram of the method.

Data were obtained from two independent experiments (two groups of 10 mice each were used to perform the analysis in duplicate). A diagram of the method is shown in Figure 2.

### 2.8. Statistics

The obtained data were analyzed with two-tailed Student’s *t*-tests and one-way ANOVA to analyze group comparisons using GraphPad Prism^®^ software, version 7. For all statistical tests, a *p* value < 0.05 was considered significant. Sample size was calculated by risk difference, by subtracting the cumulative incidence in the non-infected animals from the cumulative incidence in the group with the infected animals.

## 3. Results

**Figure 3:**
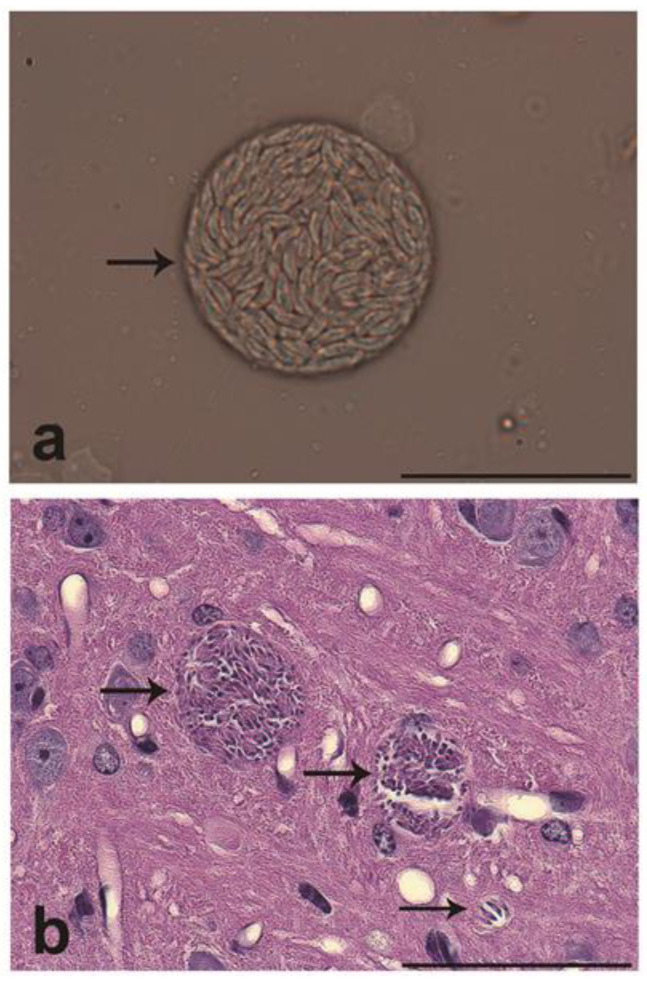
(**a**) Isolated ME49 *T. gondii* tissue cyst obtained from the maceration of a mouse infected brain. Image was taken with a 60X oil immersion objective lens. (**b**) Photomicrograph of sagittal section of cerebral cortex from infected CD-1 mouse stained with hematoxylin and eosin showing bradyzoites within tissue cysts (black arrows). Young tissue cysts can contain only two bradyzoites, whereas older ones may contain more than 1000. Bars 100 µm.

**Figure 4:**
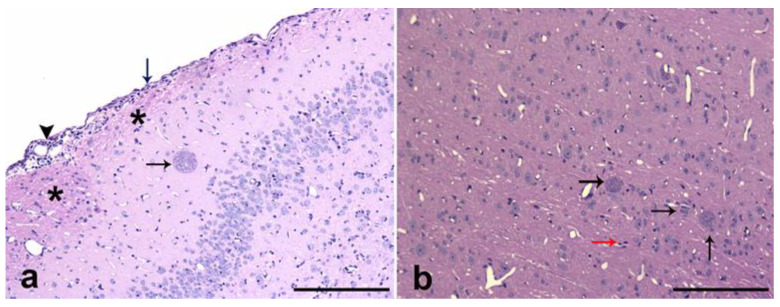
(**a**) Photomicrographs of sagittal sections of cerebral cortex from infected CD-1 mice, stained with hematoxylin and eosin. Meninge thickening can be observed (deep blue arrow) as well as mononuclear cells (black arrow), neuropile degeneration (asterisks), *T. gondii* cyst (black arrow), and diffuse gliosis. Bar 100 µm. (**b**) Photomicrographs of sagittal sections of cerebral cortex from infected CD-1 mice, stained with hematoxylin and eosin with *T. gondii* cysts (black arrows) and gliosis (red arrow). Bar 50 µm.

No signs of illness were observed in the infected mice during infection. From the homogenized infected brains, *T. gondii* cysts were isolated and observed under light microscopy (Figure 3). In the histological samples from all the infected animals, brain tissue cysts with well-defined membranes containing bradyzoites were observed. Cysts were distributed all through the brain; nevertheless, a significant selective tropism of *T. gondii* toward a particular system was not observed. The cerebral cortex was the predominant location, with a mean of 23 ± 1.51 cysts. A low incidence of tissue cysts was observed in the limbic system mostly near the lateral ventricle and less towards the hippocampus area (16 ± 1.41). Brains from the non-infected mice appeared with normal histology. Hematoxylin and Eosin sections from the infected mice showed diffuse gliosis, satellitosis, neuropile degeneration, meningeal thickening, and mononuclear cells (Figure 4). In some samples, cysts were surrounded by multiple vascular beds with a moderate reactive endothelium and some spongiosis zones. No clinical signs or brain cysts were observed in the non-infected mice.

### BrdU-Positive Cells’ Evaluation by Confocal Scanning

**Figure 5:**
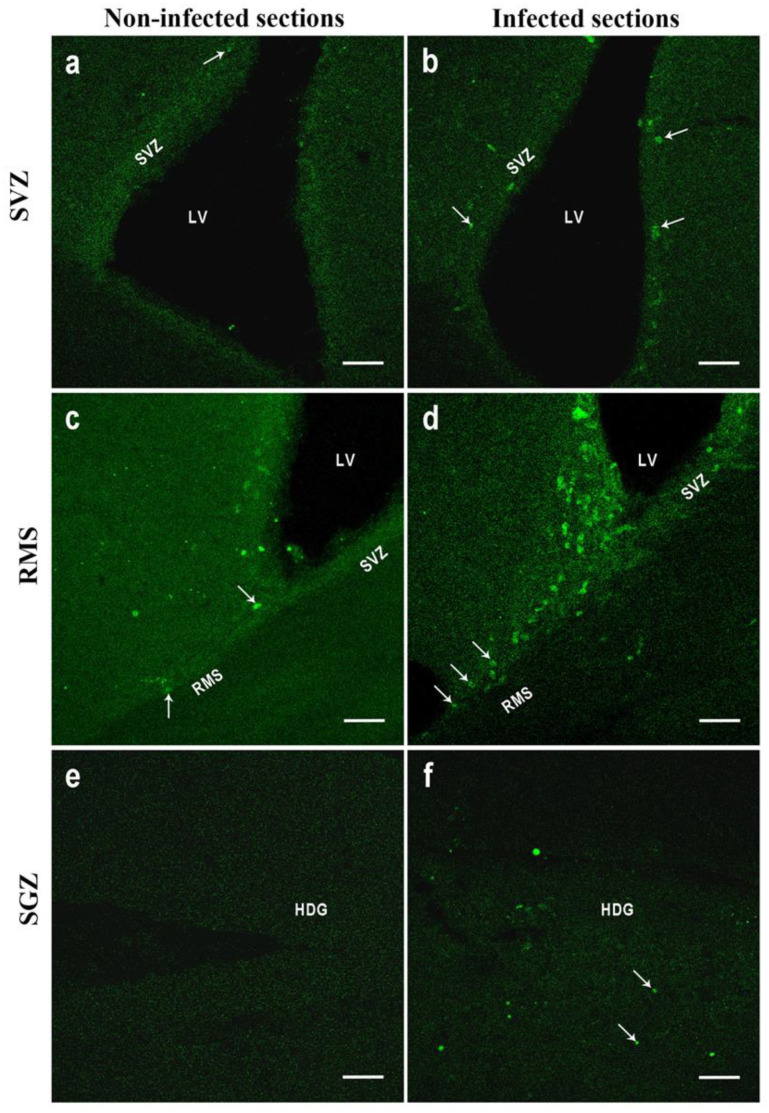
Immunofluorescence brain images of sagittal and coronal sections of the subventricular zone of the lateral ventricles, rostral migratory stream, and hippocampal dentate gyrus from infected and non-infected mice showing BrdU+ NPCs. (**a**) Non-infected subventricular zone coronal section, (**b**) infected subventricular zone coronal section, (**c**) non-infected rostral migratory stream coronal section, (**d**) infected rostral migratory stream coronal section, (**e**) non-infected dentate gyrus sagittal section, (**f**) infected dentate gyrus sagittal section. BrdU+ NPCs show an intense green fluorescence and are indicated by white arrows. SVZ (subventricular zone), LV (lateral ventricle), RMS (rostral migratory stream), HDG (hippocampal dentate gyrus). Bars 50 µm.

Results are presented as mean ± SE/mm^3^. Coronal and sagittal sections from the SGZ and SVZ as sagittal sections from the RMS of the infected and non-infected mice were evaluated in order to assess NPC proliferation. A significant increase in the proliferating cells was observed at the three evaluated regions of the infected mice in comparison with that of the control group (non-infected animals) (Figure 5). In general, NPC proliferation in the infected animals was more than triple when compared with that of the control group, with a statistical significance between *p* < 0.0001 to *p* < 0.0030 set by Prism software (significance was set at *p* < 0.05 versus control group). BrdU+ NPC proliferation in the sagittal and coronal sections of the SGZ of infected animals (10 ± 2.19 and 11.14 ± 1.14, respectively) was considerably increased in comparison to that of the non-infected group (2.12 ± 0.17; 2.12 ± 0.29, respectively). In the SVZ of infected animals, BrdU+ NPC proliferation was also significative (17.5 ± 3.93 in the coronal sections and 11.49 ± 0.99 in the sagittal sections) when compared with that of the non-infected animals (2.57 ± 0.97 and 4.02 ± 0.61 for both sections, respectively). In the sagittal sections of the RMS, a significant increment was also observed, with a mean of 14.82 ± 2.01 BrdU+ NPCs in the infected animals versus 6 ±1.06 in the non-infected mice.

**Figure 6:**
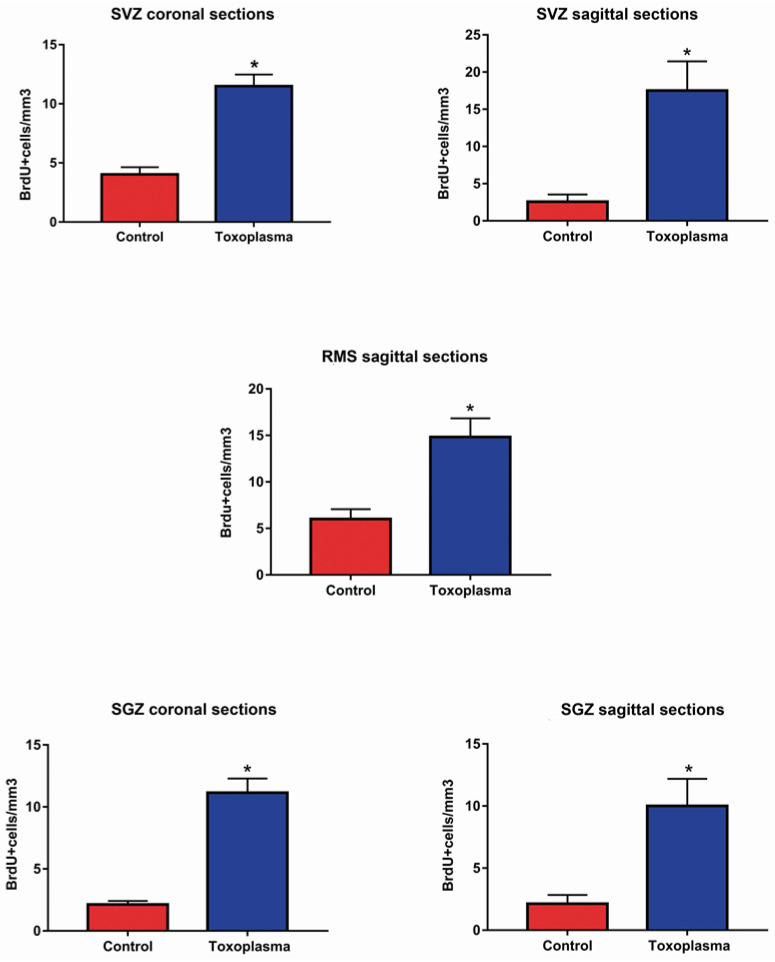
Proliferation of NPCs in coronal or sagittal sections from the SVZ, RMS, and SGZ of *T. gondii*-infected mice versus control group. Results are the mean of NPC number per mm^3^ ± stand error of two independent experiments. * *p* < 0.05 versus control group.

NPC proliferation results can be observed in Table 1 and Figure 5 and Figure 6.

## 4. Discussion

In the present study, we evaluated the proliferation of NPCs in mice harboring a *T. gondii* chronic infection in both the SVZ and SGZ, as well as the cells’ migration through the RMS, as neurogenesis takes place in these zones in the adult brain of some mammals [10,17]. Our results showed that the infected mice harbored a mean of 39 cysts, located mostly in the cerebral cortex. It has been reported by other authors that in a chronic infection with *T. gondii*, the number of developed brain cysts depends on the host susceptibility to infection and on the parasite strain, and therefore, oral infection with 10 cysts of *T. gondii* genotype II strains can result in less than 30 to more than 700 cysts per animal, regularly located in the frontal cortex and limbic area. Thus, our results are consistent with those obtained by these authors [32,35,36].

Mice susceptibility to toxoplasmosis is strain-dependent; type II strains such as ME49 display intermediate virulence in some mice such as CD-1 mice [32,37]. Nevertheless, low parasite loads can still cause inflammation, as it is now known that cysts are dynamic growing entities, despite being considered dormant and metabolically inert structures in the past [32,38]. Even a low parasite load of dynamic cysts can cause brain cell death, inflammation, and the production or modification of some cytokines [39]. In recent studies, the correlation between toxoplasma chronic infection and modifications in neurochemistry, neuronal structure, and gene expression in the host brain has been reported; even with a lack of clinical signs or other evidence of chronic infection, the presence of *T. gondii* can change the expression of some genes involved in inflammation and immune response [8,40]. Even though infected mice presented a low load of tissue cysts in the present study, some inflammatory lesions could be observed in the histological sections.

All the infected animals showed a significant increase in NPC proliferation in the three evaluated areas in comparison with those of the non-infected group. BrdU was used to detect NPC proliferation. Several studies have discredited the argument that BrdU may not detect proliferative cells but rather only DNA under repair in postmitotic neurons. Immunofluorescence and electron microscopy studies have shown that BrdU-positive cells developed into fully mature neurons. Additionally, the use of retroviral vectors (which only integrate into dividing cells) confirmed the BrdU labeling indicated adult stem cell proliferation. In cells in which DNA repair mechanisms are observed, as in apoptotic or irradiated cells, labeling with BrdU is not observed, or it is severely and instantly reduced [41,42].

It is important to know that NPCs give rise to neuronal and a few glial cell types in the adult central nervous system of some mammals, including humans [43]. In mammals, NPCs that migrate through the RMS to the OB or from the SGZ to the granular cell layer of the dentate gyrus are migratory neuroblasts that might become functional neurons if they survive. BrdU is a ‘gold standard’ by which all other markers in neurogenesis research are measured; nevertheless, by itself, BrdU does not give any indication of the phenotype of marked cells.

The role of NPC proliferation in the mammal’s brain has been studied mostly in neuronal pathologies, such as traumatic brain injury, ischemia, and behavioral disorders, as well as in some infectious diseases; for instance, neurogenesis after stroke is associated with tissue repair in mice [44]. In response to an infection, effector cells of the immune system in the brain release several inflammatory molecules, and some aspects of this neuroinflammatory response lead to beneficial or detrimental brain outcomes [45]. In neonatal rats infected with lymphocytic choriomeningitis virus, neurogenesis was impaired, probably due to a virus-induced impoverishment of dentate granule cells’ neuronal progenitors [46]. NPCs seem to be the main population affected by Zika virus, with consequent premature differentiation and defective cell division in in vitro studies [47]. HIV-1 proteins and/or secondary immune/inflammatory responses impair the initial differentiation process of NPCs [48], and *Plasmodium bergehi* Anka also impairs adult hippocampal neurogenesis [49]. Contrarily, neurogenesis induced by the enzyme Neil3, which eliminates oxidative DNA base damage, protects mice against scrapie strain RML prion disease during the clinical phase [50]. Ampicillin-sensitive commensal bacteria regulate the neurogenesis in the SVZ of the adult mouse brain, and neurogenesis and prolongevity signaling in young germ-free mice transplanted with the gut microbiota of old mice were observed in a recent study [51,52]. Neurogenesis stimulated by exercise seemed to increase survival in mice with bacterial meningitis [53,54].

In vitro studies have shown the effect of some *T. gondii* molecules that interfere with the survival of the NPCs. Excretion/secretion antigens and virulence factor ROP18 of the *T. gondii* RH strain downregulate βIII-tubulin protein in C17.2 neural stem cells, in consequence blocking their differentiation [24,55]. Zhou et al. (2015) stated that *T. gondii* induced weaker apoptosis of neural C17.2 stem cells in vitro, thus reducing their proliferation [56,57]. This experiment was performed with an atypical *T. gondii* strain (TgCtwh3) that does not produce chronic toxoplasmosis and was found to have high virulence to mice as a type I RH strain. Highly virulent strains are found to contain more ROP18 proteins that can downregulate NFκB transcription factor and induce host cell apoptosis. NFκB regulates many genes that are important in inflammation, immunity, cell survival, and neural plasticity. Therefore, NFκB signaling has been reported in initiating early proliferation and differentiation of adult and embryonic neural stem cells [58]. Likewise, Bcl-2 enhances neurogenesis and inhibits apoptosis in a murine damaged brain [59,60,61,62]. Unfortunately, our results cannot be compared with those obtained in these studies, since the conditions in our experiment were performed in vivo, and cell differentiation was not determined. Subsequently, until this study, there has been no available information regarding the proliferation of NPCs in in vivo models of *T. gondii* chronic infection. In chronic infections with *T. gondii*, different intrinsic factors that influence adult NPC proliferation can be observed, such as neuronal damage and modification of inflammatory cytokines [10].

Histopathological sections of chronic infected mice frequently reported necrosis, gliosis, infiltrated inflammatory cells, the presence of cysts (mostly in the gray matter), and neuronal degeneration [63]. In the histological sections of our infected animals, gliosis was the main observed lesion; this could therefore in part explain the proliferation of NPCs, as increasing evidence indicates that these cells may play regenerative and reparative roles in response to brain injuries or diseases [64]. Gliosis is a reaction of the central nervous system to injury of the brain and involves the proliferation of several types of glial cells, including astrocytes [63]. In brain damage, inflammation and microglial activation play a prominent role in determining the balance of NPC proliferation and impinge on the rate of adult neurogenesis according to anti-inflammatory/pro-inflammatory states [10,65,66]. It has been demonstrated that IFNγ enhances NPCs and neurogenesis in mice and protects against neuronal damage rather than exacerbating it [67]. IFNγ also enhanced neuronal differentiation directly when administered to NPCs in vitro [68,69,70,71]. Currently, *T. gondii* chronic infection has acquired more clinical importance in the immunocompetent host, since recent studies have demonstrated that encysted bradyzoites continue to replicate in vivo. Therefore, permanent glia activation is observed, as is the migration of immune cells that produce cytokines that can modify the function and structure of neurons, such as astrocytes, which may play regenerative and reparative roles in response to brain injuries or disease [64,72,73,74,75].

One limitation to our study is that we did not use different markers of undifferentiated cells to assess whether NPCs could differentiate. Consequently, it is necessary to study their neurogenic potential, as well as their impact during chronic infection and in mice behavior. This model could serve as a basis for the elucidation of neural cellular events that may take place during chronic *T. gondii* infection as well as of their relationship with the development of host behavioral alterations due to neurogenesis imbalance.

## 5. Conclusions

It is concluded that chronic toxoplasmosis can increase NPC proliferation; reactive gliosis and chronic neuroinflammation evoked by active bradyzoites could trigger this increment. Thus, the pivotal role of neurogenesis in in vivo models of *T. gondii* neuropathology and therapeutics should be examined by future studies.

## Figures and Tables

**Table 1:** Proliferation of neural progenitor cells in chronically infected *T. gondii* mice.

Brain Section	NPC Number in Control Group (Non-Infected Animals)/mm^3^	NPC Number in *T. gondii*-Infected Animals/mm^3^	Statistical Significance (*p* < 0.05)
Sagittal SGZ	2.12 ± 0.71	10.00 ± 2.19 *	0.0014
Coronal SGZ	2.12 ± 0.29	11.14 ± 1.14 *	0.0001
Sagittal SVZ	2.57 ± 0.97	17.50 ± 3.93 *	0.0018
Coronal SVZ	4.02 ± 0.61	11.49 ± 0.99 *	0.0001
Sagittal RMS	6.00 ± 1.06	14.82 ± 2.01 *	0.0030

Results are the mean of two independent experiments and standard error. *p* < 0.05 versus control group. * Statistical significance versus control group.

## Data Availability

Not applicable.
